# Grain legume cultivation and children’s dietary diversity in smallholder farming households in rural Ghana and Kenya

**DOI:** 10.1007/s12571-017-0720-0

**Published:** 2017-10-11

**Authors:** Ilse de Jager, Abdul-Razak Abizari, Jacob C. Douma, Ken E. Giller, Inge D. Brouwer

**Affiliations:** 1 Division of Human Nutrition, Wageningen University, PO Box 17, 6700, AA Wageningen, The Netherlands; 2 Plant Production Systems Group, Wageningen University, PO Box 430, 6700, AK Wageningen, The Netherlands; 3 Department of Community Nutrition, School of Allied Health Sciences, University for Development Studies, PO Box TL 1883, Tamale, Ghana; 4 Centre for Crop Systems Analysis group, Wageningen University, Droevendaalsesteeg 1, 6708, PB Wageningen, The Netherlands; 5 Laboratory of Entomology, Wageningen University, PO Box 8031, 6700, EH Wageningen, The Netherlands

**Keywords:** Dietary diversity, Legume production, SEM analysis, Children, Ghana, Kenya

## Abstract

Boosting smallholder food production can potentially improve children’s nutrition in rural Sub-Saharan Africa through a production-own consumption pathway and an income-food purchase pathway. Rigorously designed studies are needed to provide evidence for nutrition impact, but are often difficult to implement in agricultural projects.Within the framework of a large agricultural development project supporting legume production (N2Africa), we studied the potential to improve children’s dietary diversity by comparing N2Africa and non-N2Africa households in a cross-sectional quasi-experimental design, followed by structural equation modelling (SEM) and focus group discussions in rural Ghana and Kenya. Comparing N2Africa and non-N2Africa households, we found that participating in N2Africa was not associated with improved dietary diversity of children. However, for soybean, SEM indicated a relatively good fit to the posteriori model in Kenya but not in Ghana, and in Kenya only the production-own consumption pathway was fully supported, with no effect through the income-food purchase pathway. Results are possibly related to differences in the food environment between the two countries, related to attribution of positive characteristics to soybean, the variety of local soybean-based dishes, being a new crop or not, women’s involvement in soybean cultivation, the presence of markets, and being treated as a food or cash crop. These findings confirm the importance of the food environment for translation of enhanced crop production into improved human nutrition. This study also shows that in a situation where rigorous study designs cannot be implemented, SEM is a useful option to analyse whether agriculture projects have the potential to improve nutrition.

## 1 Introduction

Over two billion people suffer from multiple micronutrient deficiencies worldwide, with high prevalence among young children in sub-Saharan Africa (Muthayya et al. 2013). More than one in three children under five years of age in sub-Saharan Africa are stunted (UNICEF et al. 2015). The majority of malnourished people live in rural areas and depend on agriculture as an important source of the food and income required for their nutrition and health (Pinstrup-Andersen 2012). Agricultural interventions therefore have great potential to improve nutrition, but this potential is yet to be unleashed (Ruel and Alderman 2013). There is a strong call for evidence to support this, based on rigorous research (Masset et al. 2012).

Boosting the production of grain legumes by smallholder farmers is a feasible option to improve nutrition in rural areas. The advantage of grain legumes like cowpea, groundnut and soybean is twofold. First, legumes are unique in that they can fix nitrogen from the air in symbiosis with Rhizobium bacteria, increasing their production and enhancing soil fertility, thus increasing the production of other crops (Giller et al. 2013). Second, compared with maize, which is the most commonly produced and consumed staple in sub-Saharan Africa, legumes are better sources of high quality protein and contain a larger variety and greater concentration of micronutrients (de Jager 2013; FAO et al. 2012; Lukmanji et al. 2008).

Many agricultural interventions aim to increase food production from one or several crop(s) and assume this will result in improved nutrition outcomes. Literature describes many different potential pathways through which agricultural projects may affect nutrition outcomes positively, but also negatively (Du et al. 2015; Hoddinott 2011; Herforth and Harris 2014). The main pathways identified are: crop production for own consumption (the production-own consumption pathway), crop production for income used to purchase food (the income-food purchase pathway) and improvement of women’s status in crop production and nutrition (the women’s empowerment pathway). The production-own consumption pathway assumes that increased production of nutritious foods increases consumption of these foods and adds to diversity of the household’s diet (Du et al. 2015; Masset et al. 2012). Greater dietary diversity results in improved nutrient adequacy of the diet, which is especially important for vulnerable groups like young children (Kennedy et al. 2007; Moursi et al. 2008). Increased legume production may lead to increased consumption of legumes, adding to dietary intake of energy, proteins, minerals and B vitamins, and improved dietary diversity. In Malawi, for example, an agriculture and nutrition education project offering different legume intercrops (including groundnut and soybean) to farmers, resulted in increased cultivation of legumes, increased the frequency of legume consumption by children and improved their nutritional status in villages that were most intensely or longest involved in the project (Bezner Kerr et al. 2007; Bezner Kerr et al. 2010). The authors did not report on the impact on children’s dietary diversity. The income-food purchase pathway assumes that increased agricultural income through increased production is used for immediate or future household needs, including food and non-food purchases to support improved nutrition outcomes such as dietary diversity (Du et al. 2015). Results of studies on effects of increased income on dietary intake are inconsistent and vary per country (Keats and Wiggins 2014). Some studies found positive effects (Muhammad et al. 2011; Monteiro 2009) and others found no effects (World Bank 2007; Masset et al. 2012) or suggested negative effects as diets tend to shift from cereals and tubers to meat, fats and sugar (Keats and Wiggins 2014). The women’s empowerment pathway is a cross-cutting pathway interacting with the production-own consumption and the income-food purchase pathways. Women’s status in the household is often related to children’s dietary intake, as found in a study in Northern Ghana by Malapit and Quisumbing (2015). In the case of increased legume production, a greater status of women may lead to increased control over resources related to legume production and more income from the sale of legume produce. In turn, women’s greater control over resources may result in the channelling of nutritious foods within households to the advantage of children, and to more income spent on nutritious food and health care, particularly for children (Smith et al. 2003; UNICEF 2011). However, the increase of female participation in agriculture may trade off with time spent on care practices, negatively influencing child nutrition (Barrios 2012; Cunningham et al. 2015).

The food environment, defined as the Bcollective physical, economic, policy, and socio-cultural surroundings, opportunities, and conditions that influence people’s food and beverage choices" (Swinburn et al. 2015), is at the interface between food production and dietary intake, and includes the availability, affordability, convenience and desirability of various foods. For example, the effect of increased legume production on children’s dietary diversity may depend on the household’s landholding influencing all three pathways. The landholding of households is associated with the quantity of household crop production and the household’s agricultural income (Mather 2009). However, the food environment is often not measured in agriculture-nutrition evaluations (Herforth and Ahmed 2015). To better understand the effect of boosting food production on children’s dietary diversity, quantitative assessments of the production-own consumption and the incomefood purchase pathways are needed, while taking into account the role of women and the food environment.

More rigorous and better designed studies are needed in agriculture and nutrition evaluations (Masset et al. 2012) but these have methodological challenges such as with establishing proper comparison groups, lacking baseline data and matching the project implementation process with rigorous study designs (Menon et al. 2013). A mixed methods design is used more frequently in project evaluations as the triangulation of complementary methods may add more rigour in evaluations (Creswell and Plano Clark 2011). Structural equation modelling (SEM) compares alternative models to assess relative model fit and is a powerful robust method for modelling complex causal paths taken by mediating variables (Garson 2015). SEM has not been used in agriculture and nutrition evaluations and may be a relevant additional method to analyse the complex pathways in this field.

We studied the potential of increased household legume production to improve the dietary diversity of children in two different sub-Saharan African rural settings, Ghana and Kenya, by using a convergent parallel mixed method design (Creswell and Plano Clark 2011) to explore and differentiate the productionown consumption pathway and the income-food purchase pathway. First, we compared children’s dietary diversity of households that did or did not participate in an agricultural intervention boosting legume production, using a cross-sectional quasiexperimental study design. Second, we studied the direction, the strength and the relative importance of the production-own consumption and the income-food purchase pathways to acquire insight in how an agricultural intervention may improve children’s dietary diversity.We qualitatively studied these pathways through focus group discussions, as well as explored the potential of assessing these pathways through the quantitative method of structural equation modelling.

## 2 Materials and methods

### 2.1 Study areas

The study was carried out in Northern Ghana and in Western Kenya with widely contrasting agro-ecological characteristics. Northern Ghana has one cropping season per year of 5 to 6 months starting in May, an average annual temperature of 28 °C and annual rainfall of 900 to 1040 mm. The main crops are maize, rice, sorghum, pearl millet, soybean, cowpea, groundnut and yam. Travel time to urban markets is between 1 to 7 h and human population density is sparse with 50 to 100 inhabitants per km^2^ (Franke et al. 2011).Western Kenya has a short cropping season of 3 months from October and a long season lasting 6 months from March, an average annual temperature of 21 °C and annual rainfall of 1350 to 1800 mm. The main crops are maize, pearl millet, groundnut, tea, beans, cassava and sweet potato. Travel time to urban markets is between 1 and 5 h and population is dense with 300 to 1200 inhabitants per km^2^ (Franke et al. 2011). This study was carried out in Karaga district in Northern Region and Bawku West district in Upper East Region in Ghana, and in Western province and Nyanza province in Kenya. These two contrasting locations in Ghana and Kenya were selected because, among the N2Africa project (see next sub-section) locations in these countries, they differed most in agro-ecological characteristics and therefore were assumed to best represent Northern Ghana and Western Kenya.

### 2.2 N2Africa intervention

The study was conducted in the context of an agricultural intervention designed to boost grain legume production, the N2Africa project. N2Africa is a large scale development-to-research project that aims to enable smallholder African farmers to benefit from symbiotic nitrogen fixation by grain legumes through effective production technologies (Giller et al. 2013). Phase I of N2Africa was implemented in Ghana and Kenya from 2009 to 2013 and during that period N2Africa was not designed to be nutrition-sensitive.

Each farmer participating in N2Africa received once a package with seed of an improved legume variety, triple superphosphate (TSP) fertilizer, and in cases where soybean seeds were provided, they also received rhizobia inoculant. Each cropping season from 2009 to 2013 different farmers received a package (18000 and 20000 packages in 2010, 32000 and 55000 in 2011, 75000 and 85000 in 2012 and 2013 in Ghana and Kenya, respectively) (Woomer et al. 2014). In Ghana, farmers received improved seeds of cowpea, groundnut or soybean and in Kenya farmers received improved seeds of soybean or climbing bean. Farmers tested the package on their own fields, with different treatments of seed and fertilizer on sub-plots. In the case of cowpea and groundnut the two treatments included no inputs (control) and with TSP (treatment) for two different varieties. In case of soybean, the four treatments included no inputs (control), with TSP, with inoculants, and with both TSP and inoculants. N2Africa was implemented through groups of farmers of 30 people (in Ghana) and 20–25 people (in Kenya), consisting of a ‘lead’ farmer who was trained in crop management practices directly by N2Africa and ‘satellite’ farmers who were trained by the lead farmer. In Kenya, some satellite farmers received the package twice and were referred to as ‘progressive’ farmers. Lead farmers had try-outs of 20 × 30 m with four sub-plots of 10 × 15 m and ‘satellite’ farmers had try-outs of 20 × 20 m with four sub-plots of 10 × 10 m. Training on processing of legumes, especially soybean, was received by some of the female farmers. These activities were numerous and diverse across eight N2Africa countries and due to the scale of the operation could not be systematically monitored (Woomer et al. 2014).

The training and the testing of different legume technologies on farmer’s own fields aimed to motivate farmers to subsequently adopt technologies, thereby increasing both their land under legume cultivation and legume productivity, resulting in increased legume production. In a study carried out among N2Africa participants in 2013, the majority of N2Africa participants reported an increase in legume area cultivated, in legume production and in input use compared with four years ago prior to the N2Africa intervention (Stadler et al. 2016). In Kenya, 52% reported an increase in soybean area cultivated, 81% reported an increase in soybean production and 9% reported using inoculants, 16% P fertilizer or blend and 61% both inputs (input value chains are most advanced in Kenya) after the N2Africa intervention. In Ghana, farmers reported an increase in area under soybean, cowpea and groundnut cultivation of 42%, 36% and 30%, respectively, and reported an increase in soybean, cowpea and groundnut production of 61%, 62% and 37%, respectively. Furthermore, in the case of soybean, 6% reported using inoculants, 19% P fertilizer or blend and 6% both inputs after the N2Africa intervention. For cowpea, 10% reported using P fertilizer or blend, and for groundnut, 15% reported using P fertilizer or blend after the N2Africa intervention (Stadler et al. 2016). Farmer field trials showed that the average increase in soybean, cowpea and groundnut yield after N2Africa was 350 kg/ha, 100 kg/ha and 100 kg/ha, respectively. In the case of full adoption of N2Africa practices (i.e., use of improved seeds, TSP fertilizer and, in the case of soybean, inoculants), the average increase in soybean, cowpea and groundnut yield was 800 kg/ha, 450 kg/ha and 200 kg/ha, respectively (Woomer et al. 2014).

### 2.3 Cross-sectional quasi-experimental study and structural equation modelling

#### 2.3.1 Subject selection

**Fig. 1:**
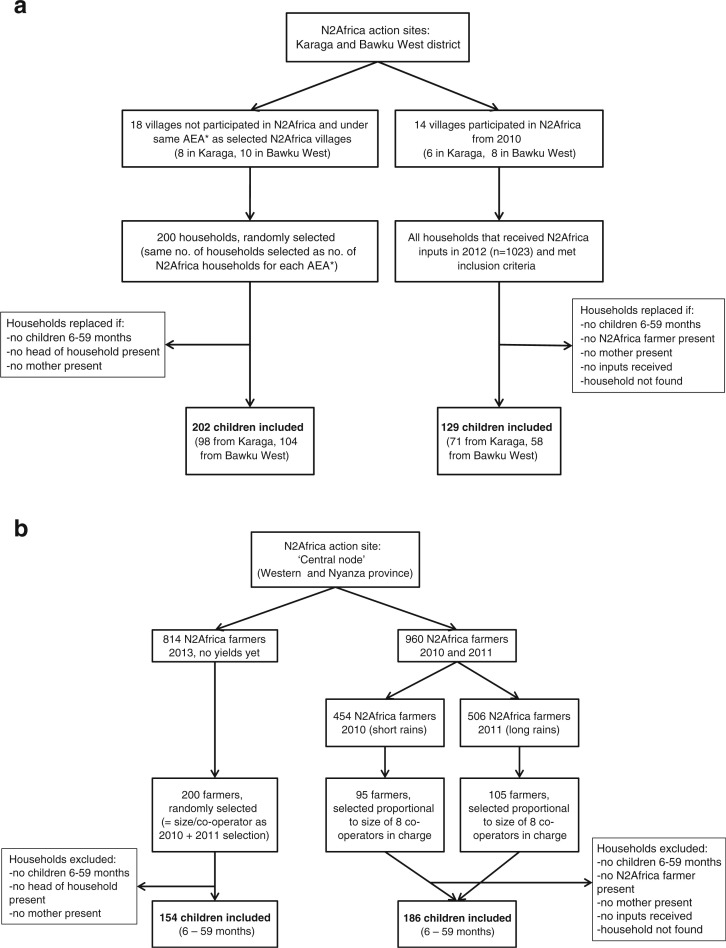
Flow chart of sample selection for the cross-sectional quasi-experimental study in Ghana a and Kenya b. N2Africa = is an agricultural project focused on putting nitrogen fixation to work for smallholder farmers growing legume crops in Africa. No. = number. AEA = agricultural extension agent. ‘Central node’ = action site of N2Africa that covers both Western and Nyanza province in Kenya. Short rain = short cropping season of 3 months fromOctober in Western Kenya. Long rain = long cropping season lasting 6 months from March in Western Kenya. Co-operators = local partners implementing N2Africa project

For the cross-sectional quasi-experimental study, infants and young children (6 to 59 months old) from households that participated in the N2Africa project (N2Africa group) and from households that did not participate in N2Africa (non-N2Africa group) were included (Fig. 1). A sample size of 400 (200/group), taking into account that 15% of households may refuse to take part in this study, was estimated to be sufficient to detect a difference in height-for-age z-scores (HAZs) of rural Ghanaian and Kenyan children (6 to 59 months old) of 0.4 and assuming an SD of 1.5 HAZ (District Monitoring and Evaluation Team Ghana et al. 1999–2001), at a 5% significance level with 80% power. Reliable estimates of expected differences in children’s dietary diversity and its distribution were not available, therefore HAZ was used as the outcome measure for the sample size calculation.

Households were included that had recently participated in N2Africa prior to data collection. For the N2Africa group in Ghana, households were randomly selected from those that received inputs from N2Africa in 2012. These were from villages that had participated in N2Africa since 2010. In Ghana, each village is linked to an agricultural extension agent and each agent has more villages under his or her supervision. For the non-N2Africa group in Ghana, all villages were selected that were under supervision of the same agricultural extension agent as the selected N2Africa villages but that did not participate in N2Africa. From these villages, households were selected by the random walk method (UN 2005). For each agricultural extension agent, the same number of households were selected for the non-N2Africa group as for the N2Africa group. For the N2Africa group in Kenya, households were randomly selected from those that received soybean inputs from N2Africa in the short rainy season in 2010 and in the long rainy season in 2011. For the non-N2Africa group in Kenya, households were randomly selected among those that received N2Africa soybean inputs in the short rainy season in 2013 but had no harvest yet at the time of data collection. In both countries, households were included when a child of 6– 59 months of age (if more than one was present, one was selected at random), mother or caregiver of the selected child and N2Africa farmer (N2Africa group) or household head (non-N2Africa group) were present. Households that did not meet these criteria were replaced randomly. For the SEM, data from the children and their households in the N2Africa and non-N2Africa group selected for the cross-sectional quasi-experimental study were combined.

#### 2.3.2 Data collection

Data were collected in the lean season in Ghana in March 2013 and in Kenya in November and December 2013 by trained interviewers who spoke the local language. Informed consent was obtained from the N2Africa farmers (N2Africa group) or household heads (non-N2Africa group).

**Household characteristics and legume production** A structured questionnaire-based interview was conducted. The N2Africa farmer (N2Africa group) or household head (non-N2Africa group) from the household of the selected child was interviewed to collect information on household composition, education, landholding, livestock ownership, assets, sources of income, labour hired-in (whether other people work on the household’s field(s), for cash or in kind), labour hired-out (whether household members work on other people’s field(s), for cash or in kind). Livestock assets recorded included cattle, donkey, pig, sheep, goat, chicken, guinea fowl, duck and dove. Tropical Livestock Unit conversion factors defined as a mature animal weighing 250 kg (Jahnke 1982) were used to calculate total livestock value in Tropical Livestock Units (TLU) in each household. Household assets included availability of a functioning radio, television, bicycle, motor, corn mill, private and/or commercial vehicle. The total value of assets in each household was calculated by the summed proportion of local market value of each available asset relative to the most expensive asset locally available. Total production of all legume crops from the previous year was recorded in local units together with the quantity used for home consumption, sold, and for other uses. Conversion factors were collected to convert local weight units to kg. In addition, specific information on participation in N2Africa was collected and also whether other legumes or legume-related and nutrition-related interventions provided outside of N2Africa were received during the last four years. The mother or caregiver of the selected child was interviewed on the child’s age and sex; and the mother’s age, education, occupation and religion.

**Children’s legume consumption and dietary diversity** A short food frequency questionnaire was administered to mothers or caregivers to collect data on the frequency of consumption of different legumes (groundnut, cowpea, soybean, Bambara groundnut, pigeon pea, climbing bean, kidney bean and mungbean) by children during the last month. Through a qualitative multi-pass 24-h-recall method (Gibson and Ferguson 2008; FAO 2010), mothers or caregivers were asked to mention all foods and beverages their child had consumed during the preceding 24-h (wakeup-to-wakeup) including anything consumed outside the home. After probing for likely-to-be-forgotten foods such as snacks and fruits, they were asked to give detailed descriptions of the foods and beverages consumed, including ingredients for mixed dishes. The 24-h-recall data were used to calculate an Individual Dietary Diversity Score (IDDS) (FAO 2010), being a count of the number of food groups consumed. Consumption of any amount of food from each food group was sufficient to ‘count’, except if an item was used as a condiment. We used the seven food groups recommended by WHO et al. (2007) that were validated to reflect nutrient adequacy of children aged 6–23 months. The seven groups included: (1) grains, roots and/or tubers; (2) legumes and/or nuts; (3) dairy products; (4) flesh foods; (5) eggs; (6) vitamin A rich fruits and/or vegetables; and (7) other fruits and/or vegetables (WHO et al. 2007). Fruits and vegetables were classified as vitamin-A rich when they provided 60 retinol activity equivalents (RAE) per 100 g or more (FAO 2010), using the Tanzania Food Composition data base (Lukmanji et al. 2008) for Kenya and the Mali (Barikmo et al. 2004) and West African Food Composition data base (FAO et al. 2012) for Ghana. Consumption of four or more food groups out of these seven is associated with better quality diets of infants and young children of 6–23 months (Working Group on Infant and Young Child Feeding Indicators 2007). Mean IDDS was calculated for all children and separately for children aged 6–23 months and children of 24–59 months. For children of 6–23 months, the proportion of children who had a nutrient diverse diet (IDDS= > 4) was calculated.

**Children’s nutritional status** Weight and length or height of children were measured following standard procedures (Cogill 2003). Weight was measured with an electronic scale to the nearest 0.1 kg (UNIscale: Seca GmbH, Hamburg, Germany). Height and length was measured with a UNICEF wooden three piece measuring board with a sliding foot or head piece and with a precision of 0.1 cm. Children below 24 months old or who were not able to stand were measured lying down (length). Children aged 24–59 months were measured standing up (height). Both length/height and weight were measured twice for each child and the average of the two measurements was taken. Scales were calibrated with a standard weight each day of data collection. Age was calculated using the date of birth from verifiable documents (health record, weighing card, birth certificate) or estimated based on a traditional calendar or another record (29 children in Ghana and 36 children in Kenya) and the date of the survey. Height and weight measurements were converted into height-for-age, weight-for-height and weight-for-age z-scores using theWHO Child Growth Standards (WHO Multicentre Growth Reference Study Group 2006) by using the WHO SPSS syntax (WHO 2011). Children who were more than two standard deviations below the reference median of height-for-age, weight-for-height and weight-for-age z-scores were classified to be stunted, wasted and underweight, respectively.

### 2.4 Focus group discussions

**Fig. 2:**
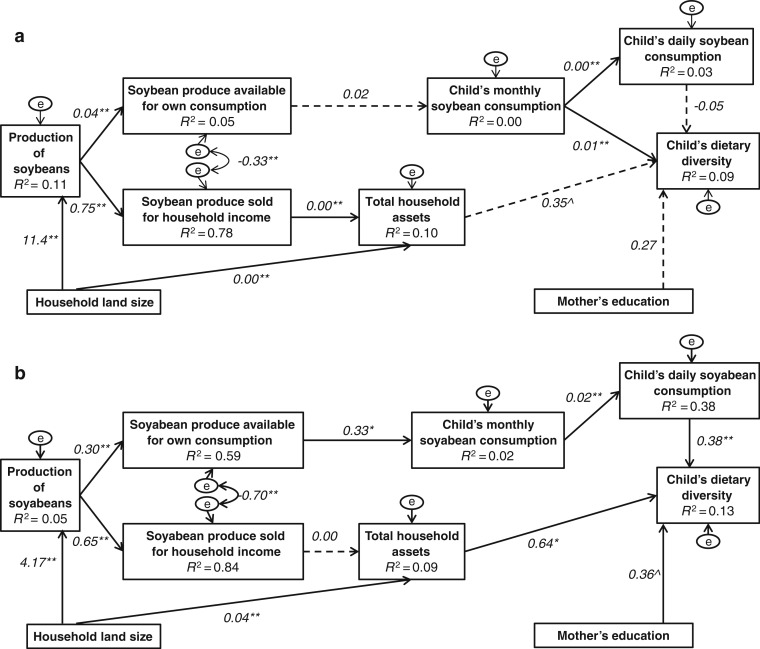
Explorative structural equation model of the effect of soybean production on dietary diversity of children 6–59 months of age through the production-own consumption pathway and income-food purchase pathway in rural Northern Ghana (n = 260) **a** and in rural Westerm Kenya (n = 197) **b**. Ghana **a:** X^2^(df) = 62.13 (24), P = 0.00 and Kenya **b:** X^2^(df) = 22.59 (24), P = 0.64 (corrected with Bollen-stine bootstrap). Values are unstandardized regression coefficients (^P < 0.10, *P < 0.05, **P < 0.01, path coefficients not significantly different from zero are shown by broken lines). Value between error terms of soybean yield available for own consumption and for household income is the estimated correlation. Part of the variance explained by the model (R2) is given under the variable names. ‘e’ is the unexplained variation. Appendix 2 shows cases excluded. Appendix 3 specifies indicators used in model. Appendix 4 and 5 provide the co-variance matrix for Fig. 2a and 2b, respectively

Both in Ghana and in Kenya, eight focus group discussions were held, four among female farmers and four among male farmers who participated in the N2Africa project. The discussions were held close to the homes of selected participants and lasted between 1 and 2 h. The discussion was led by a researcher and supported by a trained local translator. Qualitative in-depth information was collected on the production-own consumption and income-food purchase pathways for grain legume production (with a focus on soybean) (Fig. 2). The theoretical pathways were used as a topic guide for the discussions. We recorded all discussions and translated and transcribed all the records into English.

### 2.5 Ethical considerations

The study was not subjected to review by a Research Ethics Board. It was part of a development project where participants were included based on implementer preferences and the willingness of participants to participate and did not include random allocation to either the intervention or control group. Approval for the study was obtained from the District Assembly, District Ministry of Agriculture offices and leaders of selected communities. Participation was voluntary and written informed consent was obtained from caregivers of selected children, with thumb prints used for those who were not literate. The identity of the infants and their mothers/caregivers has been kept confidential.

### 2.6 Statistical analysis

Statistical analyses were performed using SPSS (IBM SPSS Statistics 22). Data were checked for normality by visual inspection of histograms and Q-Q plots. Non-normal data were log- or square root-transformed to approach normality. Accordingly, geometric means with 95% confidence intervals (CI) are presented. Two approaches were used to study the potential effect of enhanced grain legume production. First, univariate statistics were applied to test for differences in the characteristics between the non-N2Africa and N2Africa groups. Second, to explore interdependencies of the variables under study, the data of both the non-N2Africa and N2Africa group combined were used for SEM.

Differences in characteristics between the non-N2Africa and N2Africa groups were analysed with independent T-test (for continuous data), and Chi-Square test (for categorical data) using a post hoc test (adjusted standardized residuals and Bonferroni correction (Beasley and Schumacker 1995)) where the independent variable had more than two categories. Twosided P < 0.05 was regarded as statistically significant.

To quantify and disentangle the various pathways from legume production to children’s dietary diversity, SEM (Garson 2015) was used for data on soybean production (targeted by N2Africa in both countries). Path analysis is a technique to explicitly test multivariate causal relations between variables. It tests the likelihood of observing the data given a set of causal relations between household characteristics and the children’s dietary diversity. We posited that through the production-own consumption pathway enhanced soybean production in the household (kg) would result in an increased quantity of soybean produce used for home consumption (kg). In turn, an increased quantity of soybean produce used for home consumption should result in increased children’s monthly soybean consumption (times per month), increasing children’s daily consumption (times per day) of soybean and enhancing children’s dietary diversity (IDDS with range of 0 to 7 food groups). In addition, children’s daily soybean consumption (times per day) should positively affect IDDS. We posited that through the income-food purchase pathway, enhanced soybean production in the household (kg) results in an increased quantity of soybean produce sold (kg), increased quantity of soybean produce sold results in increased income (total value of household assets), and increased income results in improved children’s dietary diversity (IDDS with range of 0 to 7 food groups). Further, we hypothesized that the quantity of soybean produce used for home consumption depends on quantity of soybean produce sold and vice versa. We also hypothesized that larger household land size (ha) results in more soybean production, thereby affecting both pathways. Finally, we posited that enhanced women’s status (mother’s education, low or high level) will result in improved children’s dietary diversity (Fig. 2). Studies show that mother’s schooling reduces the risk of stunted children (Ruel and Alderman 2013) and therefore education is often used as an indirect measure of women’s status. The degree of fit of the hypothesized models to the data was measured by comparing the observed and measured covariance matrices. To account for non-normality of the data, a bootstrap derived chi-square statistic was used (Bollen-Stine bootstrap; 2000 samples). Lack of significant fit (P > 0.05) means that the hypothesized model is rejected as a causal explanation of the data. All individual relationships were tested for significance using z statistics. The SEM was performed using AMOS, an add-on module for SPSS (IBM SPSS Amos 23.0.0).

All transcripts from the focus group discussions were read thoroughly several times, focusing on one theme (one of the steps in the pathways), at a time. Key words and phrases were underlined, categorized per theme and separated for women and men participants. Given the objective of this study, the convergence and inconsistencies per theme were classified. This thematic analysis gave insight into which steps of the pathways were or were not present and which factors influenced the absence of steps, according to most participants.

## 3 Results

### 3.1 Characteristics of children, their mothers and households

In Ghana, 202 versus 126 children, and in Kenya 154 versus 186 children, were included in the non-N2Africa group and the N2Africa group, respectively (Fig. 1). Characteristics of the children, their mothers and households in the non-N2Africa and N2Africa groups were comparable in both countries (Table 1). Ghanaian children were on average 29 months old and Kenyan children 34 months old, with about half being female in both countries. In Ghana and Kenya, the percentage of stunted and wasted children in the non-N2Africa and N2Africa groups did not differ. Chronic and acute malnutrition were more prevalent among Ghanaian children than Kenyan children (32% versus 24% stunted children and 9.4% versus 5.3% wasted children, respectively, P < 0.05). In both countries the majority of the mothers of the selected children were farmers. In Ghana more mothers had no education compared with Kenyan mothers (83% versus 15%, P < 0.05). In Ghana, but not in Kenya, we found more Muslim mothers (55.8% versus 30.3%) in the N2Africa group compared with the non-N2Africa group. Ghanaian households were comprised of more household members than Kenyan households (11.1 (10.4–11.8) versus 6.2 (6.0–6.4), respectively, P < 0.05). In Ghana but not in Kenya, households were larger (12.0 versus 10.5 household members) in the N2Africa group compared with the non-N2Africa group. Also in Kenya but not in Ghana, the child-to-adult ratio was smaller (1.5 versus 1.8) in the N2Africa compared with the non-N2Africa group. Ghanaian households owned about ten times more land than the Kenyan households (13 (12–14) ha versus 1.3 (1.2–1.4) ha, P < 0.05). In Ghana but not in Kenya, there were more households with at least one household member who had completed a higher level of education (42.6% versus 21.1%) and households had more varied sources of income (2.5 versus 2.0) in the N2Africa compared with the non-N2Africa group.

**Table 1:** Demographic and social economic characteristics of children aged 6–59 months, their mothers and their households in the non-N2Africa group and the N2Africa group in Ghana and Kenya

	Ghana		Kenya	
	Non-N2Africa (*n* = 202)^a^	N2Africa (*n* = 129)^a^ *% or (geometric) mean (95%CI)*	Non-N2Africa (*n* = 154)^a^	N2Africa (*n* = 186)^a^
Characteristics				
Children				
Age, in months	28.4 (26.5–30.2)	30.6 (28.0–33.2)	34.8 (32.5–37.1)	34.1 (31.9–36.3)
Age < 24 months, %	37.6	31.0	27.9	25.8
Females, %	48.5	54.3	54.5	48.4
Stunted (chronic malnourished), %	29.7	35.7	27.3	21.5
Wasted (acute malnourished), %	11.4	6.2	4.5	5.9
Mothers of children				
Age, in years	30.5 (29.3–31.6)	30.9 (29.6–32.3)^2^	33.2 (31.5–35.0)	32.0 (30.5–33.5)^2^
Education level^c^, %				
None	85.6	78.7	17.1	13.0
Primary education	14.4	19.7	57.9	62.7
Higher education	0.0	1.6	25.0	24.3
Occupation is farmer, %	62.9	72.4	80.4	82.8
Religion (Islam, Christian)^d^, %	30.3	55.8**	97.4	98.4
Households of children				
People in household	10.5 (9.7–11.3)	12.0 (11.0–13.4)^2^*	6.1 (5.8–6.4)	6.5 (6.2–6.8)^1^^
Child:adult ratio in household	1.3 (1.2–1.4)	1.2 (1.0–1.3)2	1.8 (1.6–2.0)	1.5 (1.4–1.7)^2^*
Highest education in hh^e^, %				
None	55.3	31.8^*^	0.0	0.0
Primary education	23.6	25.6	18.2	13.0
Higher education	21.1	42.6^*^	81.8	87.0
Total Land size (ha)	13 (12–15)	13 (11–15)+2	1.4 (1.2–1.6)	1.2 (1.1–1.3)^2^
Livestock (in TLU^f^)	3.2 (2.7–3.8)	2.9 (2.3–3.6)^2^	1.1 (1.0–1.4)	1.3 (1.1–1.4)^2^
Assets, total value in hh^g^	0.08 (0.05–0.10)	0.12 (0.08–0.16)^1^^	0.03 (0.02–0.04)	0.03 (0.02–0.04)^1^
Main source of income, %				
Cropping	83.2	79.1	75.3	67.2
Livestock	12.9	15.5	1.3	3.2
Other^h^	4.0	5.4	23.4	29.6
Number of income sources^i^	2.0 (1.8–2.1)	2.5 (2.3–2.7)^2^**	2.3 (2.2–2.4)	2.4 (2.3–2.5)^2^^
Labour hired-in^j^, %				
None	16.0	11.6	45.5	46.5
In kind	50.0	42.6	6.5	2.7
For cash	34.0	45.7	48.1	50.8
Labour hired-out^k^, %				
None	8.0	7.0	37.0	42.2
In kind	69.7	69.8	9.7	4.3
For cash	22.4	23.3	53.2	53.5

^*^P < 0.05, ^**^P < 0.01 (comparing N2Africa and non-N2Adrica within countries)

^1^ square root transformation, ^2^ log10 transformation

a See Appendix 1 for missing data per variable and group

b Values are percentage, geometric mean (95%CI) or mean (95%CI). Type of transformation applied is indicated for geometric values

c Highest completed education of the mother: none (none or primary school not completed), primary education (primary school and/or literacy class or Arabic school completed) or higher education (secondary and/or tertiary education completed)

d Percentage of mothers who are Islamic in Ghana and who are Christian in Kenya (major religion in area)

e Highest completed education in the household: none, primary education or higher education (for more details see ^c^)

f Tropical livestock Units (TLU) defined as a mature animal weighing 250 kg, conversion factors: cattle (0.7), pig (0.2), sheep, goat (0.1), chicken, guinea fowl, duck, dove (0.01) (Jahnke 1982)

g Summed proportion (calculated in local market prices in Ghana Cedis and Kenyan shilling relative to most expensive asset) of assets available in household. Conversion factors for Ghana and Kenya, respectively: radio (0.001 and 0.002), TV (0.011 and 0.011), bicycle (0.013 and 0.013), motor (0.150 and 0.149), corn mill (0.210 and 0.213), private (1and 1) and commercial vehicle (1 and 1)

h Other sources of income include casual labour, trade, other business, salaried job, remittances and pension

i Total number of different sources of income

j Other people work on the household’s field(s): none, in kind or for cash

k Household members in the household who work on other people’s field(s): none, in kind or for cash

### 3.2 Participation in N2Africa and other interventions

Of all N2Africa farmers included in this study, 77.3% were satellite farmers and 22.7% were lead farmers in Ghana while 48.9% were satellite farmers, 50.0% were ‘progressive’ farmers and 1.1% were lead farmers in Kenya. In Ghana 39.5% and in Kenya 71.0% of the participants were female. In Ghana most of the farmers received soybean (74.4%), some cowpea (25.6%) and a few groundnut (2.3%) seeds. More than half of farmers reported to have received fertilizer (60.5%) and about half of farmers who reported to have received soybean seeds said they also received inoculant (38%). In Kenya, all farmers reported to have received soybean seeds and almost all also reported to have received fertilizer (92.9%) and inoculant (91.3%). In both Ghana and Kenya, it was reported that others in their household had received support from N2Africa in the same and/or previous season, respectively 29.5%and 37.7%. In Ghana 96.1%and in Kenya 44%of all farmers reported to have received training from N2Africa in crop management practices and/or training on soybean processing. In Ghana, the training received was mainly related to management practices while in Kenya training was mainly on soybean processing.

Subjects from the non-N2Africa and N2Africa groups reported to have received other legume, legume-related, (human) nutrition and/or nutrition-related interventions provided outside of N2Africa during the last four years. In Ghana and in Kenya, the number of subjects from the non-N2Africa and N2Africa group that reported to have received nutrition and nutritionrelated education received outside of N2Africa did not differ (11.4% and 7.4% in Ghana, 2.6% and 1.1% in Kenya). In Ghana but not in Kenya, more subjects from the N2Africa group reported they had received legume or legume-related interventions provided from outside of N2Africa compared with the non-N2Africa group (14.8% versus 5.9%).

### 3.3 Household legume production

In Ghana and in Kenya, the total household production of all grain legumes was comparable in the two groups (Table 2). However, the proportion of households cultivating legumes was greater in the N2Africa group compared with the non-N2Africa group (100% versus 88.1% in Ghana and 100% versus 94.8% in Kenya, respectively). In Ghana, less of total household legume production was used for home consumption than in Kenya (37% versus 65% of production, P < 0.05). In Ghana but not in Kenya, less of total household legume production was used for home consumption in the N2Africa households compared with the non-N2Africa group (29% versus 43%). Different results were found for the individual grain legumes. In Ghana and in Kenya, more N2Africa households cultivated soybean compared with the non-N2Africa group (90.7% versus 75.2% and 94.1% versus 18.2%, respectively) but among the farmers who grew soybean the total production of soybean and percentage used for consumption or sold did not differ between groups in both countries. In the case of cowpea, in Ghana fewer households in the N2Africa group cultivated cowpea compared with the non-N2Africa group (40.3% versus 55.4%) and less of the cowpea production was used for consumption (52% versus 69%), with no differences between groups in Kenya. Total production and percentage sold did not differ for cowpea between the non-N2Africa and N2Africa groups in Ghana and Kenya. In both Ghana and Kenya, the proportion of households that cultivated groundnut and that cultivated other legumes not received from N2Africa, their total production, and their percentage sold did not differ between the non-N2Africa and N2Africa groups. This was also the case for the percentage of production used for home consumption, except for groundnut in Ghana where fewer households used them for consumption in the N2Africa group compared with the non-N2Africa group (3% versus 7%).

**Table 2:** Cultivation of grain legumes, their total production and percentage consumed or sold^a^ reported in the non-N2Africa group and the N2Africa group in Ghana and in Kenya

	Ghana		Kenya	
	Non-N2Africa (*n* = 202)^b^	N2Africa (*n* = 129)^b^ % *or (geometric) mean(95%CI)*^c^	Non-N2Africa (*n* = 154)^b^	N2Africa (*n* = 186)^b^
Soybean				
Households cultivated, %	75.2	90.7^**^	18.2	94.1^*^
Yield of 0^d^, %	1.3	2.6	7.1	1.7^
Household production (kg)^e^	271 (218–337)	257 (194–340)^2^	13 (9–21)	9 (7–10)^2^^
Consumed (%)^f^	15 (10–20)	10 (6–15)^1^	64 (52–76)	65 (60–70)
Sold (%)^f^	32 (25–41)	30 (23–39)^1^	23 (12–33)	22 (18–26)
Cowpea				
Households cultivated, %	55.4	40.3^**^	8.4	13.4
Yield of 0^d^, %	3.6	3.8	0.0	8.0
Household production (kg)^e^	82 (63–106)	73 (49–109)^2^	6 (3–10)	5 (3–8)^2^
Consumed (%)^f^	69 (62–77)	52 (40–64)^*^	50 (28–72)	73 (60–87)^
Sold (%)^f^	24 (17–31)	27 (16–37)	20 (0–40)	9 (2–15)
Groundnut^g^				
Households cultivated,	51.0	54.3	36.4	34.4
Yield of 0^d^, %	1.9	1.4	1.8	6.3
Household production	460 (366–577)	584 (410–830)^2^	14 (10–20)	12 (9–17)^2^
Consumed (%)^f^	7 (4–10)	3 (2–5)^2^*	66 (58–75)	74 (67–81)
Sold (%)^f^	51 (43–59)	55 (45–65)	22 (15–30)	17 (10–23)
Other legumesh				
Households cultivated, %	53.5	46.3	94.2	90.9
Yield of 0^d^, %	1.9	0.0	0.7	0.0
Household production (kg)^e^	73 (56–95)	69 (49–98)^2^	20 (17–24)	16 (14–19)^2^^
Consumed (%)^f^	76 (69–83)	70 (58–82)	66 (61–70)	69 (65–73)
Sold (%)^f^	8 (3–12)	9 (3–16)	18 (14–22)	15 (11–18)
All legumes^i^				
Households cultivated, %^h^	88.1	100^**^	94.8	100^**^
Yield of 0^d^, %	0.0	3.1^*^	0.7	0.0
Household production (kg)^e^	495 (396–620)	501 (371–677)^2^	26 (22–32)	28 (24–33)^2^
Consumed (%)^f^	43 (37–48)	29 (23–35)^**^	65 (61–69)	65 (62–69)
Sold (%)^f^	40 (34–45)	43 (37–49)	18 (15–22)	21 (17–24)

^*^P < 0.05, ^**^P < 0.01, ^*P* < 0.10 (comparing N2Africa and non-N2Adrica within countries)

^1^ square root transformation, ^2^ log10 transformation

a Other uses of grain legume production include: used for seeds, given back to N2Africa, stored or unknown

b See Appendix 1 for missing data per variable and group

c Values are percentage, geometric mean (95%CI) or mean (95%CI). Type of transformation applied is indicated for geometric values

d Percentage of households who cultivated soybean but had no yield

e Total yield in kg of previous year reported by farmers who did cultivated specific legume, excluding cases with no yield

f Mean of percentage of total yield used for home consumption or sold

g Reported shelled yield in kg is conversed to unshelled yield by conversion factor 0.4. If not indicated yield was assumed as unshelled

h Reported other legumes (not received from N2Africa) cultivated. In Ghana: pigeonpea and Bambara groundnut. In Kenya: mung bean and bush bean

i All legumes cultivated per household summed

### 3.4 Children’s legume consumption and dietary diversity

In both Ghana and Kenya, children’s monthly frequency of consumption of soybean, groundnut, cowpea and other legume varieties not distributed through N2Africa did not differ between the non-N2Africa and N2Africa groups, except that Ghanaian children’s monthly frequency consumption of cowpea was greater in the N2Africa group than in non-N2Africa group (12.6 versus 9.8, respectively) (Table 3). Compared with Kenyan children, the monthly frequency of soybean consumption was greater among children in Ghana (30.5 (26.4–35.0) versus 6.4 (5.3–7.8) times, P-value <0.05). However, 24-h-recall data showed that in Ghana soybean was consumed mostly as a condiment and not in large portions. After excluding condiment-consumption of soybean, the monthly frequency of consumption of all legumes by children in Ghana was still greater than in Kenya (61.1 (54.9–67.7)) versus 22.1 (19.6–24.9) times per month, respectively, P < 0.05). Also the daily frequency of children’s legume consumption in Ghana was greater than in Kenya (1.5 (1.3–1.7)) versus 0.3 (0.2–0.4) times per day, P < 0.05). In Ghana but not in Kenya, the daily overall consumption of legumes by children was more frequent in the N2Africa group than in the non-N2Africa group (1.9 (1.6–2.2)) versus 1.4 (1.2–1.6) times per day, P < 0.05).

**Table 3:** Reported times of soybean, groundnut, cowpea and other grain legumes consumed per month of children 6–59 months by their mother or care-giver in the non-N2Africa group and the N2Africa group in Ghana and in Kenya

	Ghana		Kenya	
	Non-N2Africa (*n* = 202)	N2Africa (*n* = 129) *geometric mean (95%CI)*	Non-N2Africa (*n* = 154)	N2Africa (*n* = 186)
Legume consumption, (times/month)^a^				
Soybean	30.8 (25.4–36.8)	30.0 (23.8–36.9)^1^	5.7 (4.1–7.7)	7.2 (5.5–9.2)^2^
Groundnut	26.7 (22.6–31.1)	30.8 (25.3–36.9)^1^	0.3 (0.1–0.6)	0.1 (0.0–0.3)^1^
Cowpea	9.5 (7.9–11.4)	12.6 (10.3–15.3)^2^*	n/a	n/a
Other legumes^b^	8.9 (7.2–11.0)	10.0 (7.8–12.7)^2^	10.0 (8.7–11.5)	8.9 (7.6–10.5)^2^
All legumes^c^	97.3 (85.3–110.0)	103.5 (89.4–118.6)^1^	21.9 (18.4–26.2)	22.3 (18.9–26.3)^2^
All legumes without soybean^d^	58.3 (50.3–66.7)	65.8 (56.0–76.4)^1^	-	-

^*^P < 0.05, ^*P* < 0.10 (comparing N2Africa and non-N2Adrica within countries)

^1^ square root transformation, ^2^ log10 transformation

a Reported times of legume consumption during the last month of a child 6–59 months of age by the mother or caregiver

b Other legumes, not received from N2Africa. In Ghana: pigeonpea and Bambara groundnut. In Kenya: mung bean and bush bean

c All legumes consumed summed

d All legumes consumed summed without soybean in Ghana. In Ghana soybean is mostly used as a condiment

In both countries, almost all children consumed grains, roots and/or tubers (94.6% versus 93.8% in Ghana and 99.4% versus 99.5% in Kenya, in the non-N2Africa and N2Africa groups respectively) and fruits and vegetables (83.7% versus 89.1% in Ghana and 100% versus 94.6% in Kenya, in the non-N2Africa and N2Africa groups) (Table 4). In Ghana and also in Kenya, the proportion of children who consumed dairy products, meat foods and eggs was similar in the non-N2Africa group compared with the N2Africa group. In Ghana (but not in Kenya), more children in the N2Africa group consumed legumes, nuts and seeds than in the non-N2Africa group (86.8% versus 77.2%, respectively) and oils and fats (79.1% versus 62.9%), but fewer consumed fruits and vegetables rich in vitamin A (34.1% versus 47%). In Kenya (but not in Ghana), fewer children consumed fruits and also vegetables in the N2Africa group compared with those in the non-N2Africa group (94.6% versus 100%).

**Table 4:** Percentage of children 6–59 months who consumed a specific food groups in the non-N2Africa group and in the N2Africa group in Ghana and in Kenya

	Ghana		Kenya	
	Non-N2Africa (*n* = 202)	N2Africa (*n* = 129)	Non-N2Africa (*n* = 154)	N2Africa (*n* = 186)
Food group	%			
1. Grain, roots and tubers	94.6	93.8	99.4	99.5
2. Legumes, nuts and seeds	77.2	86.8^*^	40.3	42.5
3. Dairy products	20.3	20.9	68.8	67.7
4. Flesh foods	87.1	89.1	36.4	32.8
5. Eggs	1.5	0.8	1.9	2.2
6. Vitamin A rich fruits and vegetables	47.0	34.1^*^	76.6	76.9
7. Other fruits and vegetables	83.7	89.1	100	94.6^*^
Oils and fats^a^	62.9	79.1^*^	97.4	94.1

* *P* < 0.05 (comparing N2Africa and non-N2Adrica within countries)

a Oils and fats are not included in individual dietary diversity score

Dietary diversity of children in the non-N2Africa group and the N2Africa group did not differ (Table 5). This was also the case for children below 24 months of age, children above 24 months and children who were not breastfed. However, dietary diversity was less among breastfed children in the N2Africa households than in the non-N2Africa group (3.7 versus 4.2, respectively) in Kenya, but not in Ghana. The percentage of children who had an IDDS of 4 or above among children below 24 months (reflecting a nutrient adequate diet) was similar in the N2Africa group compared with the non-N2Africa group in Ghana (60.0% and 65.8%) and also in Kenya (62.5% and 76.7%).

**Table 5:** Individual dietary diversity score (IDDS) of children 6–59 months in the non-N2Africa group and the N2Africa group in Ghana and in Kenya

Characteristics	Ghana		Kenya	
	Non-N2Africa (*n* = 202)^a^ *Mean (SD) or %*	N2Africa (*n* = 129)^a^	Non-N2Africa (*n* = 154)^a^	N2Africa (*n* = 186)^a^
IDDS (7 food groups, 0 to 7)^b^				
*All children*	4.1 (1.4)	4.2 (1.3)	4.2 (0.9)	4.2 (1.0)
children age 6–23 months	3.5 (1.7)	3.2 (1.7)	4.1 (0.9)	3.8 (1.2)
children age 24–59 months	4.5 (0.9)	4.6 (0.8)	4.3 (0.9)	4.3 (0.9)
*Children receiving breastmilk, %*	*42.5*	*38.1*	*24.3*	*22.2*
children non-breastfed	4.4 (0.9)	4.6 (0.8)	4.2 (0.9)	4.3 (0.9)
children breastfed	3.7 (1.7)	3.4 (1.6)	4.2 (0.8)	3.7 (1.1)^*^
Minimum dietary diversity, IDDS > =4^c^
children age 6–23 months, %	65.8	60.0	76.7	62.5

* *P* < 0.05 (comparing N2Africa and non-N2Adrica within countries)

a See Appendix 1 for sample size per group: children age 6–23 months, children age 24–59 months, children non-breastfed and children breastfed

b IDDS is computed by sum of seven food groups being consumed: 1. Grains, roots and tubers, 2. Legumes, nuts and seeds, 3. Dairy products, 4. Flesh foods, 5. Eggs, 6. Vitamin A rich fruits and vegetables and 7. Other fruits and vegetables (WHO et al. 2007)

c An individual dietary diversity score of 4 or more in infants and young children reflect a nutrient adequate diet (WHO et al. 2007)

We found no associations between demographic and socioeconomic characteristics of households (household’s highest completed education level, mother’s education level, household size, landholding, livestock, household’s assets and number of income sources) and nutrition indicators for the children, either in the N2Africa or in the non-N2Africa groups.

### 3.5 Production-own consumption pathway and income-food purchase pathway

In Ghana the hypothetical model of the production-own consumption pathway and the income-food purchase pathway was not consistent with the data (*X*^2^(df) = 62.13 (24), *P* = 0.00) (Fig. 2a), while in Kenya the hypothetical model was consistent with the data (*X*^2^(df) = 22.59 (24), *P* = 0.64) (Fig. 2b). In Ghana, both pathways included non-significant paths. In Kenya, there was only a small positive indirect effect of soybean production on the dietary diversity of children through the production-own consumption pathway, but there was no effect of soybean production on children’s dietary diversity through the income-food purchase pathway. The effect of soybean production on the IDDS was very low: a multiplication of individual path coefficients showed that an increase of soybean production by 1 kg led to an increase of 0.00075 IDDS points. Therefore to have a meaningful effect on children’s IDDS an increase in household’s soybean production of at least 1000 kg is needed. Based on soybean production of 800 kg/ha after full adoption of N2Africa interventions (Woomer et al. 2014), an increase of 1000 kg means expansion of 1.2 ha under soybean cultivation. This is highly unlikely, especially in Kenya where the average land size of a household is 1.3 ha. However, children’s monthly soybean consumption was not directly related with children’s dietary diversity. Household land size was positively related with the production of soybean and total household assets in both models, but mother’s education was not related with children’s dietary diversity (*P* = 0.06) in the Kenyan model.

In focus group discussions in both Ghana and Kenya, female N2Africa participants more commonly referred to the production-own consumption pathway and males to the income-food purchase pathway with regard to enhanced soybean production. Comparing Ghanaian and Kenyan N2Africa participants, Ghanaian participants referred less to the production-own consumption pathway but rather referred more to the income-food purchase pathway for enhanced production of soybean. In Ghana, few comments were made about soybean consumption and these comments were mixed: ‘my children are a bit more healthy because they like to eat soybean’ but also ‘I have not seen any direct effect of soybean consumption on my health’. By contrast in Kenya, participants were overall positive about soybean consumption: ‘it makes children strong’, ‘soy is so sweet’ and ‘their health has changed to good health’. In both Ghana and Kenya, participants reported they had received training on soybean processing and learned how to use soya in their local dishes. Further, Ghanaian participants were positive about the soybean market in Northern Ghana (‘it gives more money than maize’ and ‘if your yield is a lot, then you can sell to get money’) while Kenyan participants mentioned that there was no market for soybean (‘price for soybean is less than for maize’ and ‘it is difficult to sell soybean’). The remarks on the income-food purchase pathway were not consistent in both countries. The ‘extra’ income was said to be spent in a wide range of different ways, including ‘to pay school fees’, ‘household items’, ‘hire people to work on their land’, ‘buy more food’, ‘to buy fertilizer’, ‘to buy seeds’, ‘for pressing needs’ and ‘to save for the purchase of a motorbike’. Some farmers mentioned the income was used to buy more food but they did not mention whether they buy nutritious foods and whether this improved their children’s diet. Also income was spent on school fees or seeds that theoretically may have an indirect effect on human nutrition, but it remains unclear whether this was the case.

## 4 Discussion

We found no association between participating in this agricultural intervention designed to boost grain legume production and the dietary diversity of children based on a cross-sectional quasi-experimental study. SEM indicated a relatively good fit to the posteriori model in Kenya but not in Ghana, and in Kenya only the production-own consumption pathway for soybean was fully supported, with no effect through the incomefood purchase pathway. Focus group discussions showed that the Ghanaian and Kenyan context of soybean production and consumption differed in the attribution of positive characteristics, variety of local soybean-based dishes, it being a relatively new crop, involvement of women in soybean cultivation, presence of markets, and being treated as a food or cash crop.

### 4.1 N2Africa and children’s nutrition outcomes

More households were cultivating grain legumes, especially soybean, in the N2Africa group (100% in Ghana and in Kenya) than in non-N2Africa group (88.1% in Ghana and 94.8% in Kenya) but we found no differences in total grain legume production among the households cultivating legumes between the two groups in neither Ghana nor Kenya. The absence of differences in grain legume production might be due to weak implementation of the N2Africa intervention in Ghana and weak adoption of N2Africa in Kenya. In Ghana only 60.5% of participating farmers reported to have received fertilizer and less than half of farmers who received soybean seeds reported they had received inoculant. In Kenya, farmers selected for this study receivedN2Africa soybean inputs in the short cropping season (from October) in 2010 and/or in long cropping season (from March) in 2011 while the legume production data collected for this study included production from the short cropping season in 2012 and long cropping season in 2013. Therefore the effect of the N2Africa intervention in Kenya on the amount of household legume production depended on the degree of adoption of improved production technologies after participating in N2Africa. Adoption may have been restricted as the current availability of rhizobial inoculants in Africa is limited (Ronner et al. 2016), as is the availability and affordability of fertilizers and good quality seeds for rural smallholder farmers. N2Africa participants, both in Ghana and Kenya, reported in the focus group discussions that there was indeed a restricted availability of promoted N2Africa inputs and that fertilizers were too expensive. Contrary to our findings, a previous study conducted across eight countries (including Ghana and Kenya) found that N2Africa participants reported increased grain legume production (Stadler et al. 2016).

We found no differences in children’s nutrition outcomes between the non-N2Africa and N2Africa groups in Ghana and Kenya, both not in frequency of consumption of the targeted grain legumes nor in diversity of the diet. Our findings are in line with earlier findings from reviews (Masset et al. 2012; Girard et al. 2012) that suggest there is limited evidence of agricultural interventions having significant positive impacts on child nutrition. Other studies (Berti et al. 2004; Pandey et al. 2016) found that without additional programming in other areas relevant for positive nutrition outcomes, such as gender or nutrition, agricultural programs are unlikely to have a significant positive impact on nutrition. The N2Africa project did include training on soya processing and targeted inclusion of 50% female participants but during the first phase did not include nutrition-specific training or other genderrelated interventions. Also the fact that we found no differences in legume production between the non-N2Africa and N2Africa group, may explain why we found no difference in children’s nutrition outcomes. Furthermore, potential nutrition outcomes resulting from N2Africa may not be sustained from harvest until the end of the lean season, the time data were collected in Ghana and Kenya. Due to the cross-sectional nature of the study, the absence of an association between participation in N2Africa and positive child nutrition outcomes cannot be attributed to a specific cause.

### 4.2 Methodological limitations

Our study suffered from several methodological limitations that hampered our ability to detect an impact of N2Africa on human nutrition (Masset et al. 2012; Girard et al. 2012). Both the lack of detectable increased household legume production and improved children’s nutrition outcomes in the N2Africa group compared with the non-N2Africa group, could be due to the limitations related to the cross-sectional quasi-experimental study design we used. Due to the character of the intervention, we could not randomize households to N2Africa or non-N2Africa groups. Absence of randomization may cause differences between treatment groups. To overcome this problem, we matched N2Africa villages with non-N2Africa villages that were under supervision by the same agricultural extension agent in Ghana and we matched N2Africa participants with participants that had recently received N2Africa support but had not yet harvest targeted grain legumes in Kenya. Furthermore, we assumed little spill-over from the N2Africa intervention in our control groups. Comparative studies in four N2Africa countries, including Ghana and Kenya, showed that 60–100% of the farmers interviewed shared seed of soybean, cowpea and groundnut with others but very few farmers shared the key technologies of the N2Africa intervention, rhizobium inoculants and P-fertilizer (Woomer et al. 2014). The N2Africa and non-N2Africa groups seem comparable as few differences in child’s, their mother’s and household’s characteristics were found at the time of interview and detected differences in characteristics were not associated with children’s nutrition outcomes.We also do not have data at a baseline before N2Africa started for these specific villages and cannot rule out potential differences between N2Africa and non-N2Africa households before the intervention. The latent differences between the two groups and the absence of a baseline limited our ability to find differences in household grain legume production and nutrition outcomes between N2Africa and non-N2Africa groups.

In this study dietary diversity was used as a proxy for diet quality, which may also have limitations. IDDS does not differentiate among foods within a food group. This may have two consequences. First, if children already consume grain legumes, the addition of another grain legume to a child’s diet will not enhance his or her IDDS even though the added food, in our case soybean, has a better nutrient profile compared with other targeted grain legumes. Adding soybean to the diet therefore may contribute to improved nutrient adequacy of the diet but will not be reflected in an increase of IDDS in this study. However, a recent study among rural Kenyan women showed that food-based scores were only slightly more strongly associated with nutrient adequacy compared with the food group-based scores (Ngala et al. 2015). Second, if children already consume the promoted grain legume, they may consume increased amounts of this grain legume that may contribute to nutrient adequacy yet this will not be reflected in his or her IDDS. A review of dietary diversity studies suggested that scores might be improved by inclusion of portion size requirements (Ruel 2003), however, measuring portion sizes in the field is challenging (Martin-Prevel et al. 2010). Further, in our study grain legume production was selfreported by N2Africa participants (N2Africa group) and head of households (non-N2Africa group), reflecting their previous year’s produce for each grain legume individually. Selfreported measures of land size and crop production are known to be inaccurate (Carletto et al. 2013).

In addition to the methodological limitations of the current study, limitations in the design of the N2Africa project itself may have hampered the ability to find differences in children’s nutrition outcomes between the N2Africa group and non-N2Africa group as well. For a thorough evaluation of the potential nutrition impact of N2Africa, a rigorous monitoring and evaluation system needs to be in place, including indicators along the potential impact pathways towards nutrition outcomes (McDermott et al. 2015; Gelli et al. 2015). For example, no data was available for whether and which crops were replaced by improved varieties of grain legume in the N2Africa intervention, which may affect household’s overall crop diversity and the quantity of crops available in the household, and in turn may affect the diet. In case grain legume production replaces part of the maize production it may positively affect the diet while if it replaces all vegetables produced it may negatively affect the diet. Some recent studies show that improved crop diversity is positively related to improved household dietary diversity (Jones et al. 2014) but others show no relation (Rajendran et al. 2014). Limited data on intermediate indicators along the impact pathways hampered the ability to identify explanations for potential impact on nutrition outcomes.

### 4.3 Production-own consumption pathway and income-food purchase pathway

SEM indicated a relatively good fit to the posteriori model in Kenya but not in Ghana. The hypothetical model for Ghana needs improvement. In Kenya we did find an effect through the production-own consumption pathway but not through the income-food purchase pathway. Through the production-own consumption pathway in Kenya, an increase of 1000 kg of household’s soybean production may lead to a modest increase of 0.75 in IDDS. This relative high increase in soybean production is necessary because a small part of the produce may be consumed in the household and from what is consumed within the household little may end up on the plates of children. Differences in five characteristics of the food environment in Ghana compared to Kenya may explain that neither pathway was present in Ghana and only the production-own consumption pathway was present in Kenya.

First, Kenyan N2Africa participants indicated the absence of a good market for soybean while Ghanaian participants indicated there was a relatively good and stable market for soybean compared with maize. Also Kenyan participants indicated that the lack of a nearby soybean market was one of the reasons they decided not to sell their soybean produce. This explains the stronger association between increased soybean production and greater quantity of soybean used for own consumption in Kenya compared with Ghana. In Kenya, soybean was a relatively new crop while in Ghana it has been widely cultivated since the 1990s (in the non-N2Africa group by 18.2% of households in Kenya versus 75.2% in Ghana). The better established market for soybean in Ghana may have strengthened the income-food purchase pathway instead of the production-own consumption pathway.

Second, Kenyan N2Africa participant’s opinions on the taste and beliefs about potential health benefits from the consumption of soybean were overall more positive compared with those of Ghanaian participants. In Ghana, soybean was mainly consumed in the form of ‘dawadawa’, similar to a bouillon cube, and thus consumed by all household members in very small amounts. However, in contrast to Ghana, overall fewer grain legumes are consumed by infants and young children in Kenya which leaves more room for increasing the intake of soybean. In addition, Kenyan participants reported a wider variety of local dishes prepared with soybean. These factors may also have led to more soybean production for home consumption in Kenya compared with Ghana.

Third, in Ghana soybean productionwasweakly associated with the quantity of soybean used for own consumption and strongly with quantity sold, implying soybean was used as a cash crop. This result confirms statistics from the Food and Agriculture Organisation (FAO 2011). Ghanaian N2Africa households cultivated less cowpea but more soybean compared with non-N2Africa households, indicating a possible replacement of cowpea by soybean. As cowpea is mainly used for home consumption, this may suggest that increased soybean production may have led to a reduction of availability of other legume crops for home consumption.

Fourth, enhanced legume production in households where children already consume grain legumes, as in Ghana, may not affect the frequency of legume consumption and/or IDDS but may increase portion sizes consumed. Preliminary analyses from a later survey conducted in Northern Ghana in Karaga district showed that children’s daily portion sizes of cowpea, groundnut and soybean (Brouwer et al. unpublished) were associated with household’s production of these grain legumes. This suggests that an increase in household’s grain legume production may have led to the increased quantity of grain legumes consumed by children in Ghana. As the present study did not include portion sizes in the calculation of IDDS, the potential of the production-own consumption pathway may have been underestimated in Ghana.

Fifth, the proportion of female participants in N2Africa in Kenya was high (above 70%) compared to Ghana (below 40%). A stronger women’s decision-making power and control over resources like increased legume production and income from the sale of legume produce, may lead to the channelling of nutritious foods within households to the advantage of children, and to more agricultural income spent on nutritious food and health care for the family, particularly for children (Smith et al. 2003; UNICEF 2011). Female N2Africa participants indeed reported in the focus group discussions more often that the (extra) grain legume produce was used for own consumption, including their children’s consumption. In this study education was used as an indirect measure of women’s status while women’s status incorporates multiple more direct domains like decision-making-power, mobility and attitude towards domestic violence (Lee-Rife 2010; Cunningham et al. 2015). A majority of the mothers of children had not completed any form of education or only completed primary school. The absence of variation in mother’s education level may also explain the absence of an association with children’s IDDS in our study. Further, household assets were used as an indicator of household income but this may not be representative for total household income including the increased agricultural income.

An agricultural project not designed to be nutrition-sensitive that results in increased availability of a promoted food for home consumption may improve nutrition outcomes, but our findings suggest this depends on the food environment. Based on the focus group discussions and SEM analysis of the productionown consumption and income-food purchase pathways, it appears that a project such as N2Africa has more potential to improve children’s dietary diversity through the productionown consumption pathway in a context where (a) farmers attribute positive characteristics towards the targeted nutritious food, (b) a wide variety of local dishes already include the promoted food, (c) women are involved, and d) the targeted food is relatively new and considered as a food crop and not a cash crop. In addition, if there is a strong market available for the promoted food, there is a likelihood that farmers prefer to sell the promoted food instead of keeping it for home consumption. Whether this income is used for improving children’s nutrition seems unpredictable or less than expected (Herforth and Ahmed 2015). Thorough understanding of the food environment is therefore necessary to improve the nutrition-sensitivity of agricultural interventions to predict whether boosting legume production may improve the dietary diversity and nutrition outcomes of children. The cross-sectional quasi-experimental study lacked the methodologically-rigorous design needed to find and draw firm conclusions on associations. In situations where rigorous study designs cannot be implemented or are not part of project evaluation, SEM in a mixed method design is a useful option to analyse whether agriculture projects have the potential to translate in improved nutrition. To our knowledge, our study was the first to use SEM in analysing the theoretical pathways from crop production to improved human nutrition in an explorative way. Further experimental studies are needed to confirm the direction and strength of the identified individual relationships between components within the pathways.

## Open Access

This article is distributed under the terms of the Creative Commons At tribution 4.0 International License (http://creativecommons.org/licenses/by/4.0/), which permits unrestricted use, distribution, and reproduction in any medium, provided you give appropriate credit to the original author(s) and the source, provide a link to the Creative Commons license, and indicate if changes were made.
