# Supplementary material for: Grain legume cultivation and children’s dietary diversity in smallholder farming households in rural Ghana and Kenya
Source: Food Secur. 2017 Oct 11;9:1053–71. doi: 10.1007/s12571-017-0720-0 (PMC7473086; doi:10.1007/s12571-017-0720-0)
Supplement: Supplementary file 1 [file FS-2017-s12571-017-0720-0-S1.docx]

## Appendix 1 Number of missing data cases per table or figure, variable and group

| **Table** | **Variable** | **Ghana** | | **Kenya** | |
| --- | --- | --- | --- | --- | --- |
|  |  | Non-N2Africa | N2Africa | Non-N2Africa | N2Africa |
| ***No. of missing cases*** | |  |  |  |  |
| Table 1 | Mother’s age | 3 | 1 | 4 | 2 |
|  | Mother’s education level | 1 | 2 | 2 | 1 |
|  | Mother’s occupation | 0 | 0 | 1 | 0 |
|  | Mother’s religion | 1 | 0 | 0 | 0 |
|  | People in household | 0 | 1 | 0 | 0 |
|  | Household’s highest education | 3 | 0 | 0 | 1 |
|  | Household ‘s total land size | 1 | 1 | 1 | 0 |
|  | Household’s livestock | 0 | 1 | 0 | 0 |
|  | Household’s total assets | 0 | 1 | 0 | 0 |
|  | Household’s labour import | 2 | 0 | 0 | 1 |
|  | Household’s labour export | 0 | 0 | 0 | 1 |
| Table 2 | Cowpea yield, uses | 0 | 0 | 1 | 0 |
|  | Total production of groundnut | 0 | 0 | 0 | 1 |
|  | Groundnut yield, uses | 0 | 0 | 0 | 2 |
|  | Cultivation of other legumes | 0 | 21 | 0 | 0 |
|  | Total production of other legumes | 2 | 0 | 0 | 0 |
|  | Other legumes, uses | 0 | 0 | 3 | 1 |
|  | All legumes, uses | 0 | 0 | 3 | 1 |
| Table 5 | Children receiving breastmilk | 2 | 3 | 2 | 1 |
| ***Sample size per group (N)*** | |  |  |  |  |
| Table 5 | Children age 6-23 months | 76 | 40 | 43 | 48 |
|  | Children age 24-59 months | 126 | 89 | 111 | 138 |
|  | Children non-breastfed | 115 | 78 | 115 | 144 |
|  | Children breastfed | 85 | 48 | 37 | 41 |
