# Supplementary material for: Grain legume cultivation and children’s dietary diversity in smallholder farming households in rural Ghana and Kenya
Source: Food Secur. 2017 Oct 11;9:1053–71. doi: 10.1007/s12571-017-0720-0 (PMC7473086; doi:10.1007/s12571-017-0720-0)
Supplement: Supplementary file 2 [file FS-2017-s12571-017-0720-0-S2.docx]

## Appendix 2 Cases excluded for structural equation modelling

| ***Figure*** | **Country** | **Cases excluded** | **No. cases excluded** | **N**  **model** |
| --- | --- | --- | --- | --- |
| *Figure 3* | Kenya | Households no soybean cultivation | 137 | 197 |
|  | (*n*=340) | Households no yield | 5 |  |
|  |  | Households missing information on mother’s education | 1 |  |
| *Figure 4* | Ghana | Households no soybean cultivation | 62 | 260 |
|  | (*n*=313) | Households no yield | 5 |  |
|  |  | Households missing information on mother’s education | 1 |  |
|  |  | Households missing information on total land size | 1 |  |
