# Supplementary material for: Grain legume cultivation and children’s dietary diversity in smallholder farming households in rural Ghana and Kenya
Source: Food Secur. 2017 Oct 11;9:1053–71. doi: 10.1007/s12571-017-0720-0 (PMC7473086; doi:10.1007/s12571-017-0720-0)
Supplement: Supplementary file 3 [file FS-2017-s12571-017-0720-0-S3.docx]

**Appendix 3** Indicators used for structural equation modelling

| **Model variables** | **Indicators** |
| --- | --- |
| Production of soybeans | Total reported soybean production (in kg) |
| Soybean yield available for own consumption | Reported soybean production used for own consumption (in kg) |
| Soybean yield sold for household income | Reported soybean production sold for household income (in kg) |
| Total household assets | Value of total household assets available in the household (summed proportion (calculated in local market prices in Ghana Cedis and Kenyan Shilling relative to most expensive asset) of assets available in household, for specific conversions see Table 1) |
| Child’s monthly soybean consumption | Child’s frequency of soybean consumption per month (times/month) |
| Child’s daily soybean consumption | Child’s frequency of soybean consumption per day (times/day) |
| Child’s dietary diversity | Child’s individual dietary diversity score (out of 7 food groups, WHO) |
| Household land size | Total land size owned by household (ha) |
| Mother’s education | Mother of child completed a form of education (no=0, yes=1) |
