# Supplementary material for: Grain legume cultivation and children’s dietary diversity in smallholder farming households in rural Ghana and Kenya
Source: Food Secur. 2017 Oct 11;9:1053–71. doi: 10.1007/s12571-017-0720-0 (PMC7473086; doi:10.1007/s12571-017-0720-0)
Supplement: Supplementary file 4 [file FS-2017-s12571-017-0720-0-S4.docx]

## Appendix 4 Co-variance matrix for structural equation modelling, Ghana (*n*=260)

|  | Production of soybeans | Soybean yield available for own con-sumption | Soybean yield sold for household income | Total household assets | Child’s monthly soybean con-sumption | Child’s daily soybean con-sumption | Child’s dietary diversity | Household land size | Mother’s education |
| --- | --- | --- | --- | --- | --- | --- | --- | --- | --- |
| Mean (SD) | 557 (670) | 63 (116) | 373 (568) | 0.21 (0.39) | 44 (40) | 0.2 (0.5) | 4.1 (1.4) | 19 (20) | 0.2 (0.4) |
| Production of soybeans | 449842.62 |  |  |  |  |  |  |  |  |
| Soybean yield available for own consumption | 17696.60 | 13475.79 |  |  |  |  |  |  |  |
| Soybean yield sold for household income | 335865.63 | 3154.08 | 323006.24 |  |  |  |  |  |  |
| Total household assets | 57.42 | 2.74 | 57.27 | .16 |  |  |  |  |  |
| Child’s monthly soybean consumption | -8652.73 | 200.35 | -6893.36 | -1.43 | 1593.89 |  |  |  |  |
| Child’s daily soybean consumption | -24.24 | 6.66 | -32.31 | .00 | 3.53 | .24 |  |  |  |
| Child’s dietary diversity | 51.17 | 16.32 | 4.34 | .04 | 14.52 | .02 | 1.85 |  |  |
| Household land size | 4323.84 | -42.15 | 3587.15 | 2.09 | -108.07 | -.04 | -1.05 | 380.39 |  |
| Mother’s education | -32.05 | 2.79 | -28.71 | -.01 | .13 | .01 | .03 | -.37 | .13 |
