# Supplementary material for: Grain legume cultivation and children’s dietary diversity in smallholder farming households in rural Ghana and Kenya
Source: Food Secur. 2017 Oct 11;9:1053–71. doi: 10.1007/s12571-017-0720-0 (PMC7473086; doi:10.1007/s12571-017-0720-0)
Supplement: Supplementary file 5 [file FS-2017-s12571-017-0720-0-S5.docx]

## Appendix 5 Co-variance matrix for structural equation modelling, Kenya (*n*=197)

|  | Production of soybeans | Soybean yield available for own con-sumption | Soybean yield sold for household income | Total household assets | Child’s monthly soybean con-sumption | Child’s daily soybean con-sumption | Child’s dietary diversity | Household land size | Mother’s education |
| --- | --- | --- | --- | --- | --- | --- | --- | --- | --- |
| Mean (SD) | 17 (31) | 9 (12) | 7 (22) | 0.08 (0.23) | 23 (29) | 0.4 (0.9) | 4.2 (1.0) | 1.5 (1.7) | 0.9 (0.3) |
| Production of soybeans | 976.63 |  |  |  |  |  |  |  |  |
| Soybean yield available for own consumption | 296.63 | 152.43 |  |  |  |  |  |  |  |
| Soybean yield sold for household income | 630.91 | 143.01 | 485.17 |  |  |  |  |  |  |
| Total household assets | 0.21 | 0.28 | -0.15 | 0.05 |  |  |  |  |  |
| Child’s monthly soybean consumption | 128.28 | 50.27 | 74.38 | 0.47 | 845.41 |  |  |  |  |
| Child’s daily soybean consumption | 3.47 | 1.51 | 1.77 | 0.01 | 16.23 | 0.83 |  |  |  |
| Child’s dietary diversity | 2.59 | 0.98 | 1.00 | 0.04 | 2.80 | 0.25 | 0.96 |  |  |
| Household land size | 11.83 | 5.01 | 5.89 | 0.11 | 5.66 | 0.23 | 0.37 | 2.83 |  |
| Mother’s education | -0.78 | -0.28 | -0.43 | 0.01 | -0.77 | -0.01 | 0.05 | -0.05 | 0.12 |
